# A Case of Post‐Transplantation Lymphoproliferative Disorder Following Kidney Transplantation

**DOI:** 10.1155/crin/5917161

**Published:** 2026-01-08

**Authors:** Punam Ajay Raval, John Otieno Odhiambo, Hanika Patel, Ahmed Sokwala

**Affiliations:** ^1^ Department of Internal Medicine, The Aga Khan University Hospital, Nairobi, Kenya, aku.edu; ^2^ Department of Radiology, The Aga Khan University Hospital, Nairobi, Kenya, aku.edu; ^3^ Department of Nephrology, The Aga Khan University Hospital, Nairobi, Kenya, aku.edu

## Abstract

This was a case of a 65‐year‐old gentleman known to have diabetes mellitus and hypertension since 2012 and post renal transplantation in 2016. He had also been treated for hepatitis C infection in the past. His regular medications included nebivolol 5 mg once a day, tacrolimus 4 mg twice a day, mycophenolate mofetil 500 mg twice a day, prednisolone 5 mg once a day, and insulin–novomix 14 units in the morning and 6 units at night. He presented to a tertiary teaching hospital in Kenya in October 2023 with abdominal pain, vomiting, and constipation on and off for 6 months, worse in the month prior to presentation. His examination was positive for dehydration, abdominal distension, and generalized abdominal tenderness. He had a normal hematological profile and renal function. A CT scan of the abdomen showed features of small bowel obstruction from the distal ileal to distal jejunal bowel loops. The patient underwent an exploratory laparotomy with intraoperative findings of a midjejunal tumor completely obstructing the lumen with proximal dilatation. The tumor was subsequently excised. Histological specimen confirmed diffuse large B‐cell lymphoma (DLBCL) as his final diagnosis.

## 1. Background

Post‐transplant lymphoproliferative disorders (PTLDs) are important malignancies after solid organ transplantation (SOT) [1] and arise from excessive B‐cell proliferation caused by weakened immune surveillance. B cells can become infected with Epstein–Barr virus (EBV) either through viral reactivation after transplantation, primary EBV infection via the transplanted organ, or environmental exposure. Most PTLD cases (over 85%) typically occur within the first year following transplantation. PTLD caused by T‐cell proliferation is rarer and is usually EBV negative [2]. Lymphoma accounts for 21% of all malignancies in SOT recipients as compared to 4% and 5% in immunocompetent individuals, respectively, in men and women [3]. Clinically, PTLD may manifest either as localized lesion or as systemic disease. Tissue diagnosis (histopathology) is essential for PTLD diagnosis, in addition to a clear evidence of EBV DNA, RNA, or protein material [1].

## 2. Case Presentation

This was a case of a 65‐year‐old gentleman known to have diabetes mellitus and hypertension since 2012 and post right renal transplantation in 2016 due to hypertensive nephropathy, with prior history of treatment of hepatitis C. He presented to the hospital in October 2023 with a 6‐month history of insidious abdominal pain, vomiting, progressive loss of appetite, and constipation. His past medical history was remarkable for being treated with antibiotics and symptomatic relief as an outpatient in peripheral facilities. At presentation, he was dehydrated with a pulse rate of 89 bpm and raised blood pressure of 153/86 mmHg. Systemic exam was positive for abdominal distension and generalized tenderness of the abdomen. Bowel sounds were present. The initial laboratory parameters are highlighted (Table 1).

**Table 1:** Laboratory parameters.

Laboratory test	Results	Reference range
White blood cells (total)	4.37 × 10^9^/L	4–10
Hemoglobin	11.2 g/dL	13–18
Platelet count	259 × 10^9^/L	150–400
Creatinine	116 µmol/L	62–115
Potassium	3.78 mmol/L	3.5–5.5
Urea	4.6 mmol/L	3.2–8.2
Tacrolimus level	11.25 ng/mL	5–8

### 2.1. Imaging

**Figure 1:**
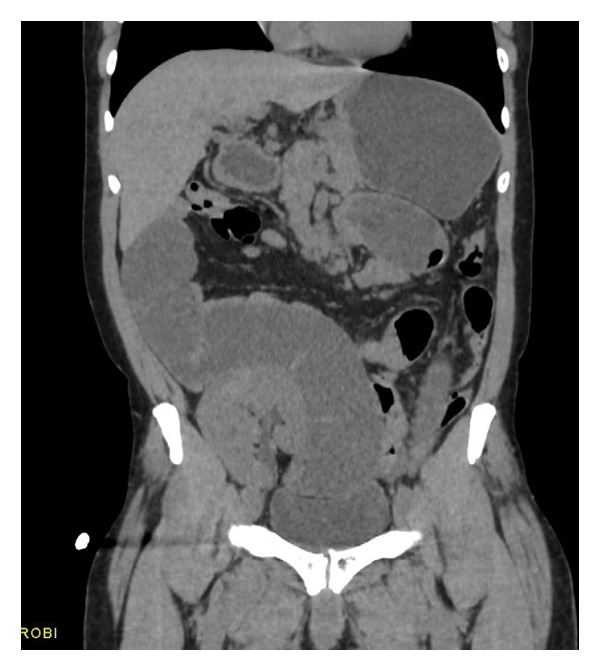
Unenhanced CT of the abdomen: coronal image at the level of the transplant kidney demonstrating dilated fluid‐filled loops of small bowel measuring up to 4.4 cm in diameter.

**Figure 2:**
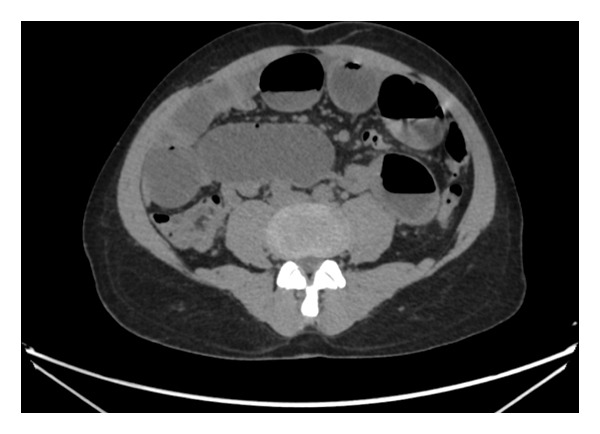
Unenhanced CT of the abdomen: axial images at the level of the mid‐abdomen demonstrating dilated fluid‐filled loops of small bowel.

Computed tomography of the abdomen showed multiple dilated fluid‐filled loops of small bowel seen from the distal jejunal to distal ileal bowel loops measuring up to 4.4 cm across their widest diameter (Figures 1 and 2). Multiple transition points were seen, one approximately 9 cm from the ileocecal junction and the rest around the right transplanted kidney. Proximal jejunum, distal terminal ileum, and large bowel were collapsed. The transplanted kidney was seen in the right iliac fossa. Stable renal cortical cyst was seen in the graft kidney measuring 1.2 cm. Mild caliectasis was seen in the transplanted kidney. No renal mass or calculi was seen in the graft kidney. Grafted ureter was normal in its course. Note was made of bilateral native atrophic kidneys. No renal masses were seen within them.

An impression of intestinal obstruction likely due to mass in a diabetic, hypertensive patient post renal transplant was made.

### 2.2. Management

**Figure 3:**
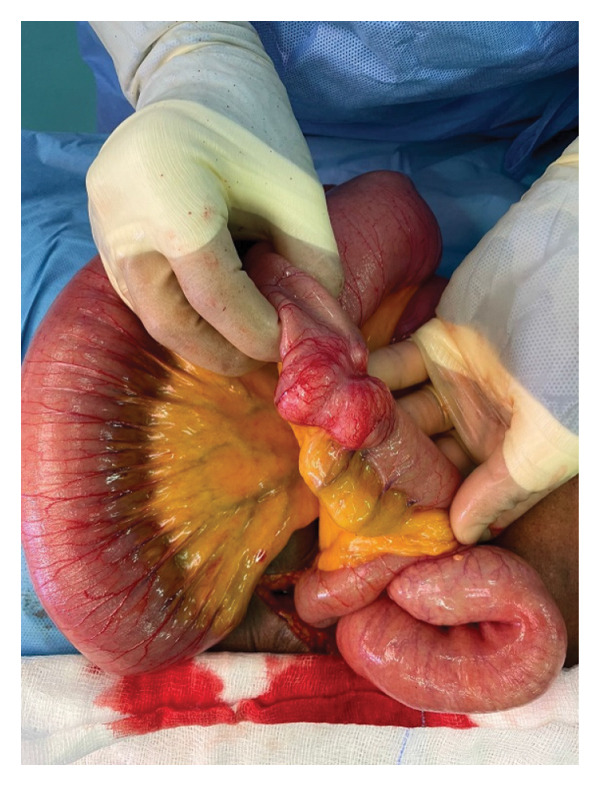
Intraoperative findings.

**Figure 4:**
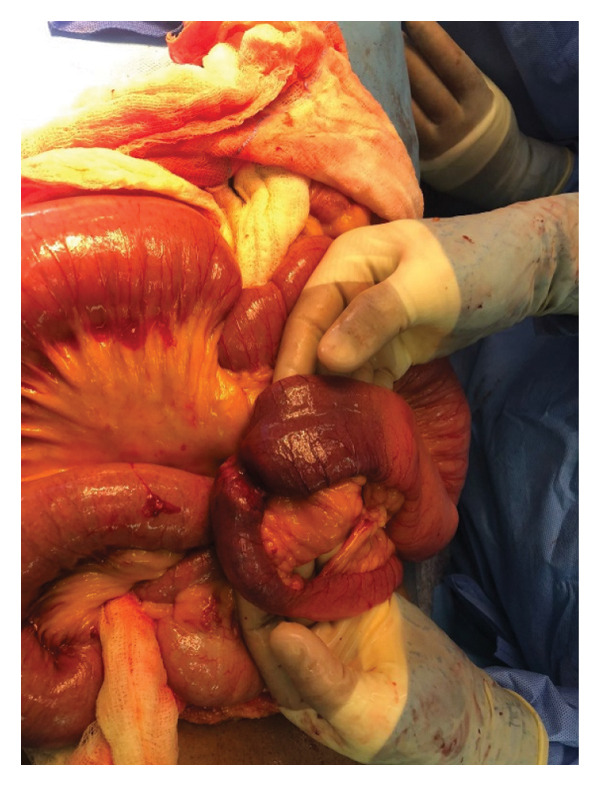
Intraoperative image demonstrating a focal segment of edematous small bowel.

He was admitted to the ward where he was commenced on intravenous fluids, analgesia, and antibiotics. He was reviewed by the surgical team and consequently underwent an exploratory laparotomy which revealed an approximately 5 × 6 cm mid jejunal tumor completely obstructing the lumen with proximal dilatation, collapsed distal jejunum, and transverse colon (Figures 3 and 4). A lymph node was noted on the small bowel mesentery close to the fourth part of the duodenum. The mass was excised and specimen wassent for histopathological review.

### 2.3. Intraoperative Findings

Intraoperative findings are present in Figures 3 and 4.

### 2.4. Histopathology

**Figure 5:**
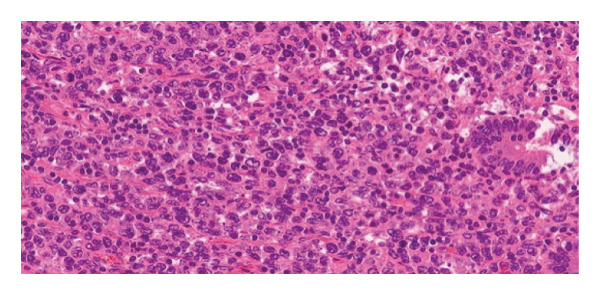
Hematoxylin‐ and eosin‐stained section showing diffuse infiltration of large atypical lymphoid cells consistent with diffuse large B‐cell lymphoma.

Sections submitted as tumor, stained using hematoxylin and eosin (H&E) staining (Figure 5), showed a diffuse infiltrate of large atypical lymphoid cells, extending beyond the muscularis propria, without serosal surface involvement. Mitoses were present and brisk. All margins were uninvolved by tumor, with a distance to closest margin measured at 3 cm.

**Figure 6:**
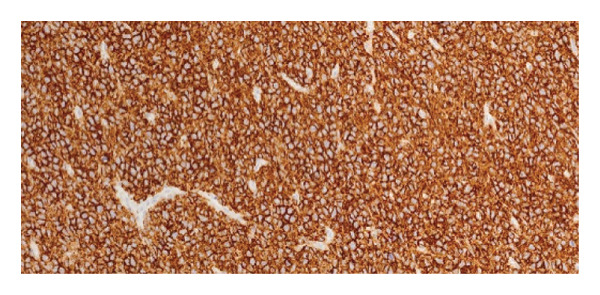
Immunohistochemistry showing diffuse membranous positivity for CD20 in tumor cells.

**Figure 7:**
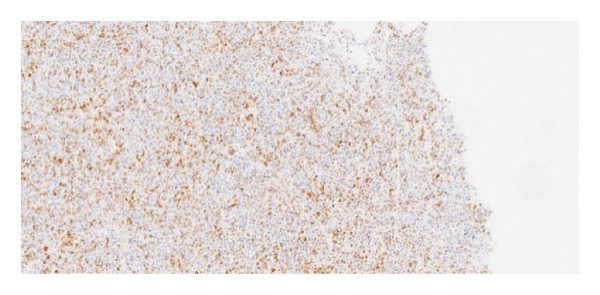
Immunohistochemistry demonstrating BCL2 positivity in neoplastic lymphoid cells.

**Figure 8:**
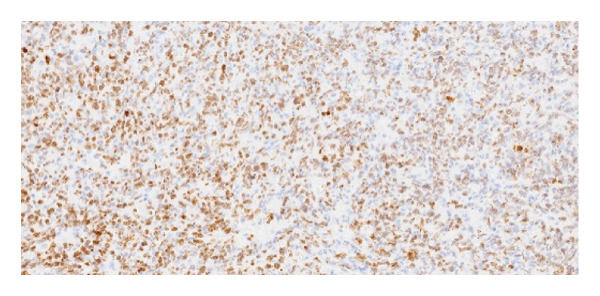
Ki‐67 immunostaining showing a proliferative index of approximately 41%–50%.

Immunohistochemical staining demonstrated the following profile: CD20: positive (Figure 6); BCL2: positive (Figure 7); CD10: negative; and Ki‐67 proliferative index: 41%–50% (Figure 8). EBV serologies were negative, and the biopsy showed no morphological features suggestive of EBV‐associated PTLD. EBER in situ hybridization was not performed.

On diagnosis of PTLD, the patient’s immunosuppression was modified: tacrolimus dose was reduced, aiming for a lower therapeutic trough while maintaining low‐dose prednisolone prior to systemic chemotherapy. In the Department of Hematology, the patient received 3 doses of R‐CHOP combination therapy. After three cycles of treatment, a complete remission was achieved, confirmed by a follow‐up PET CT scan. The patient was carefully followed up under the following schedule: every 3 months for 1 year, every 6 months for 2 years, and then once a year.

## 3. Discussion and Conclusion

PTLD is a serious, life‐threatening complication in organ transplant patients receiving immunosuppressive therapy [4]. EBV is a key factor in the development of PTLD and is present in over 90% of patients with the disorder, typically due to immunosuppressive therapy. The risk of developing lymphoproliferative disorders increases with the intensity of immunosuppression and the total dose received [5]. PTLD can present with a range of clinical courses, sometimes mimicking infectious mononucleosis with symptoms like fever, sore throat, enlarged tonsils, and lymphadenopathy [6]. In more severe cases, it can progress rapidly, leading to severe metabolic acidosis, organ dysfunction (including the graft and other internal organs), and infiltrates that may appear as tumors [6].

A higher risk of PTLD development occurs mainly in the first year after transplantation [6]. Opelz and Döhler studied more than 50,000 patients after heart or renal transplant and found a significantly higher incidence of B‐cell PTLD in the first year after transplantation, while T‐cell PTLD is expected 5 years after transplant [7].

Diagnosing PTLD requires careful consideration, as the disease often develops gradually and occurs in extranodal sites. The diagnosis is confirmed through histopathological examination of a lymph node or tissue sample from the affected organ obtained via biopsy. Additionally, demonstrating the immunoreactivity of EBV proteins in lymphocytes can be useful for diagnosis [8].

This case represents late‐onset PTLD occurring several years after kidney transplantation. Although most PTLD occurs within the first year, late‐onset disease may be associated with prolonged immunosuppression, older recipient age, prior chronic viral infections, and higher cumulative calcineurin inhibitor exposure; these factors may impair EBV‐specific immunity and permit clonal B‐cell proliferation many years after transplant [9]. In our patient, tacrolimus trough at presentation was 11.25 ng/mL, which may have contributed to disease evolution.

Management of PTLD requires a staged, risk‐adapted approach. Reduction of immunosuppression is the initial recommended step to attempt immune reconstitution. For B‐cell PTLD, rituximab monotherapy is commonly used as first‐line systemic therapy; R‐CHOP or other chemoimmunotherapy regimens are indicated for aggressive monomorphic disease or when rapid cytoreduction is required [10]. Intestinal PTLD frequently presents with complications (obstruction, bleeding, and perforation), and surgical resection plays an integral role in such cases. In our patient, surgical resection was required due to complete luminal obstruction and subsequent R‐CHOP was given for systemic disease control, resulting in complete remission.

The prognosis of PTLD depends on factors such as the type, malignancy characteristics, and disease stage. Mortality is high in monoclonal malignant forms of PTLD, reaching up to 80%. Polymorphic forms generally have a better outlook than monomorphic ones. Negative prognostic factors include high‐grade lymphoma histology, advanced disease according to the Ann Arbor classification, involvement of the central nervous system and bone marrow, a disseminated disease (affecting at least two extranodal sites), hypoalbuminemia, hepatitis B or C infection, late‐onset PTLD, age over 60, and a history of cancer prior to transplantation [11].

## Consent

All the patients allowed personal data processing and informed consent was obtained from all individual participants included in the study.

## Conflicts of Interest

The authors declare no conflicts of interest.

## Funding

No funding was received for this manuscript.
